# The Role of Urban Wastewater in the Environmental Transmission of Antimicrobial Resistance: The Current Situation in Italy (2010–2019)

**DOI:** 10.3390/microorganisms8101567

**Published:** 2020-10-12

**Authors:** Francesco Triggiano, Carla Calia, Giusy Diella, Maria Teresa Montagna, Osvalda De Giglio, Giuseppina Caggiano

**Affiliations:** Department of Biomedical Science and Human Oncology, University of Bari Aldo Moro, Piazza G. Cesare 11, 70124 Bari, Italy; francesco.triggiano@uniba.it (F.T.); carla.calia@uniba.it (C.C.); giusy.diella@uniba.it (G.D.); mariateresa.montagna@uniba.it (M.T.M.); giuseppina.caggiano@uniba.it (G.C.)

**Keywords:** urban wastewater, antimicrobial resistance, ARG, ARB, sewage treatment, UWWTP, Italy

## Abstract

Scientific studies show that urban wastewater treatment plants (UWWTP) are among the main sources of release of antibiotics, antibiotic resistance genes (ARG) and antibiotic-resistant bacteria (ARB) into the environment, representing a risk to human health. This review summarizes selected publications from 1 January 2010 to 31 December 2019, with particular attention to the presence and treatment of ARG and ARB in UWWTPs in Italy. Following a brief introduction, the review is divided into three sections: (i) phenotypic assessment (ARB) and (ii) genotypic assessment (ARG) of resistant microorganisms, and (iii) wastewater treatment processes. Each article was read entirely to extract the year of publication, the geographical area of the UWWTP, the ARB and ARG found, and the type of disinfection treatment used. Among the ARB, we focused on the antibiotic resistance of *Escherichia coli*, *Klebsiella pneumoniae*, and Enterococci in UWWTP. The results show that the information presented in the literature to date is not exhaustive; therefore, future scientific studies at the national level are needed to better understand the spread of ARB and ARG, and also to develop new treatment methods to reduce this spread.

## 1. Introduction

Antimicrobial resistance (AMR) is the ability of a microorganism to withstand the action of a drug (e.g., antibiotics, antivirals); it is caused in part by the overuse of antibiotics in medical care and animal farming. AMR poses a threat to humans, animals, and the environment, and represents a serious and growing global public health problem [1,2,3]. Worldwide, 700,000 people die each year from drug-resistant diseases [4]. According to the Centers for Disease Control and Prevention (CDC), more than 2.8 million antibiotic-resistant infections occur in the United States each year, causing more than 35,000 deaths [5]. In the European Union, AMR is responsible for 25,000 deaths per year, resulting in EUR 1.5 billion annually in healthcare costs and lost productivity [3]. Recent estimates indicate AMR could cause 10 million deaths globally by 2050 [6], with costs amounting to $1.2 trillion [7].

AMR can be natural or acquired. In the first case, bacteria are intrinsically resistant to antibiotics. This resistance, independent of previous antibiotic exposure, may depend on the absence of certain cell structures (cell wall), or the reduced permeability of the external membrane (e.g., lipopolysaccharide in Gram-negative bacteria). Natural AMR can also be induced after exposure to an antibiotic by the over-expression of efflux pumps that reduce the intracellular concentration of a given drug [8,9,10].

Acquired resistance is caused by the appearance of resistant strains within an initially sensitive microbial population, and can be either chromosomal or extrachromosomal. In chromosomal resistance, resistance is generally caused by a spontaneous mutation of chromosomal genetic information and is transmitted vertically; in extrachromosomal resistance, resistance happens following the acquisition of mobile genetic elements (plasmids, transposons) through horizontal gene transfer [11,12].

Recently, the World Health Organization published a global priority list of 12 antibiotic-resistant bacteria (ARB) that pose the greatest risk to human health [13]. The main ARB associated with serious and often deadly human infections, such as bloodstream infections and pneumonia, are *Klebsiella pneumoniae* (carbapenem-resistant strain, CRKp), *Escherichia coli* (extended spectrum β-lactamase, ESBL), *Enterococcus* spp. (vancomycin resistant Enterococci, VRE), and *Staphylococcus aureus* (methicillin resistant *Staphylococcus aureus*, MRSA) [14]. Based on national surveillance data from 2017, the United States CDC estimates that AMR bacteria cause many infections and deaths annually, in particular MRSA (323,700 infections and 10,600 deaths), ESBL producers (197,400 infections and 9100 deaths), VRE (54,500 infections and 5400 deaths), and carbapenem-resistant *Enterobacteriaceae* (CRE; 13,100 infections and 1100 deaths) [5].

In recent years, researchers have investigated AMR from an environmental origin, now considered a crucial aspect for the prevention of human health risks [15,16,17,18]. Some authors state that antimicrobial resistance genes (ARG) and ARB are widely distributed in soil and animal waste [19,20], in agri-food production [21], surface waters [22], and urban wastewater treatment plant (UWWTP) effluents [23]. It is suspected that wastewater is a hot spot for ARB and ARG [24]. Antibiotics can enter the aquatic environment as a result of inadequate wastewater treatment, the disposal of unused medicines, or agricultural runoff [25,26]. Once in the environment, antibiotics can either be easily degraded or persist and accumulate. Conventional water treatment only partially removes antibiotics [27], and although their concentrations in many UWWTP effluents and surface waters are low (usually at levels of ng L^−1^ to μg L^−1^), these concentrations could promote the acquisition of new resistance. Furthermore, biological processes (oxidation tanks) promote ARB proliferation. The co-selection of ARB by heavy metals and biocides frequently present in wastewater [28] and the presence of sublethal concentrations of antibiotics can impact cell functions and antibiotic resistance [29,30]. Waterborne *E. coli* are a major reservoir of AMR, including ESBL and *K. pneumoniae* carbapenemase (CRKp) mechanisms [31].

In Italy, in 2018, the percentage of resistance to the main classes of antibiotics was higher than the European average, mainly regarding *E. coli* CREC (third generation cephalosporin resistant; 28.7% vs. 15.1%), *K. pneumoniae* (CRKp; 26.8% vs. 7.5%), Enterococci (VRE *E. faecium*; 18.9% vs. 17.3%) [32]. Moreover, *K. pneumoniae* and *E. coli* were multiresistant to at least three classes of antibiotics (fluoroquinolones, third generation cephalosporins, and aminoglycosides) in 33% vs. 11% of cases.

Existing scientific reviews provide little information on ARB and ARG in Italy before 2018; therefore, the aim of this work was to identify knowledge gaps in this topic to allow the development of strategies to reduce AMR in Italy. In particular, the objectives of this study were: (i) to detect antibiotic resistant *E. coli*, *K. pneumoniae*, and Enterococci in urban wastewater treatment plants (UWWTPs), (ii) to identify their resistance genes, and (iii) to assess the efficacy of wastewater treatment processes.

## 2. Study Design

A systematic review of all Italian full-text articles published in English and reported in the PubMed database between 1 January 2010 and 31 December 2019 was performed, with special focus on *E. coli*, *K. pneumoniae*, and Enterococci from UWWTPs in Italy (i.e., urban wastewater treatment coming from a mixture of domestic and industrial wastewater, and/or rainwater runoff pipes conveyed into sewer systems) [33]. The following keywords were used for the searches: urban wastewater, AMR, sewage treatment, and UWWTP, in combination with each other and with the word “Italy.” The exclusion criteria were as follows: articles that reported a period of study prior to 2010, non-ARB articles, letters/short communications, reviews, minireviews, duplicates, and articles that did not concern UWWTPs in Italy.

The papers were divided into three major categories: (1) phenotypic (ARB) and (2) genotypic (ARG) evaluation of resistant microorganisms, and (3) wastewater treatment processes. They were read entirely to determine the year of publication, geographical area of UWWTP, the ARB and ARG found, and the type of disinfection treatment.

## 3. Results

There were 17 papers corresponding to ARB and/or ARG from UWWTP in Italy. Table 1 shows the chronology of the considered papers, divided by geographical area. Seven studies evaluated only the phenotypic aspects, five only considered the genotypic aspects, and five evaluated both aspects. Finally, 13 out of 17 articles evaluated the effectiveness of wastewater disinfection treatments.

### 3.1. Phenotypic Evaluation

The phenotypic methods used for the detection of ARB were: disk diffusion (Kirby-Bauer test), E-test, or medium supplemented with a mixture of antibiotics at specific concentrations.

#### 3.1.1. *Escherichia coli*

Ten articles evaluated the phenotypic profiles of *E. coli*.

Turolla et al. [39] assessed the presence of *E. coli* resistant to β-lactams (ampicillin, AMP), chloramphenicol (CHL), and tetracycline (TET) in wastewater of three UWWTP located in Milano, which differed in terms of the biological process, disinfection processes, and compliance with discharge limits. Zanotto et al. [40] reported the presence of *E. coli* resistant to AMP and/or CHL in different points of a UWWTP in Milano, i.e., inflow to biological treatment, outflow from biological treatment following rapid sand filtration, and outflow from peracetic acid disinfection.

Some studies were focused on *Enterobacteriaceae* resistant to β-lactams isolated from UWWTP located in Abruzzo [41,43]. Piccirilli et al. [41] highlighted the presence of three *E. coli* strains resistant to β-lactams (AMP and cefotaxime, CTX) isolated from stream water and UWWTP effluents draining into the Fino, Tavo, and Saline Rivers of the Abruzzo region. Moreover, two *E. coli* strains were also resistant to another β-lactam (piperacillin, PIP; cefazolin, CZ), aminoglycosides (amikacin, AK) and colistin (CS). One *E. coli* strain was also resistant to carbapenems (ertapenem, ETP) and fluoroquinolones (levofloxacin, LVX). Pellegrini et al. [43] found 12 *E. coli* strains collected from three sampling sites (seven from raw water, three from water after chlorination system, and two from final effluent) of the treatment plant. All isolates were resistant to β-lactams (amoxicillin, AMX, PIP, CTX, CZ, ceftazidime, CAZ, cephalotin, CF) and to sulfonamides (trimethoprim-sulfamethoxazole, SXT); six isolates were resistant to β-lactam inhibitors (amoxicillin/acid clavulanic, AMC) and four to fluoroquinolones (LVX).

Six studies evaluated the presence of AMR *E. coli* in UWWTP located in Salerno. Two studies [24,46] showed the presence of two *E. coli* strains resistant to β-lactams (AMX) and sulfonamides (sulfamethoxazole, SMZ), isolated from effluents (activate sludge). Another study [45] highlighted the presence of *E. coli* resistant to fluoroquinolones (ciprofloxacin, CIP), to β-lactams (CEF), TET, and glycopeptides (vancomycin, VAN) selected from the effluent (activated sludge). *E. coli* resistant to AMP, CIP, and TET were isolated from effluent samples (activated sludge); [47,48] and from effluents collected downstream of the biological process and upstream of the disinfection [44].

#### 3.1.2. *Enterococcus* spp.

Two studies evaluated the phenotypic profile of Enterococci. Russo et al. [49] reported the presence of two species of Enterococci (*E. faecium*, *E. faecalis*) isolated from effluent waters of three horizontal subsurface flows of a UWWTP located in San Marco di Ganzaria (Sicilia). *E. faecalis* strains were resistant to macrolides (erythromycin), rifamycin (rifampicin, RIF), β-lactams (AMP, penicillin G), CHL, TET, and aminoglycosides (streptomycin, gentamycin, GM). Fiorentino A. et al. [48] highlighted the presence of Enterococci resistant to AMP, TET, and fluoroquinolones (CIP) coming from Sele river which receives effluents from a UWWTP in Salerno.

#### 3.1.3. *Klebsiella pneumoniae*

Two papers focused their attention on AMR *K. pneumoniae* strains. Perilli et al. [42] evaluated the presence of *K. pneumoniae* resistant to carbapenems in five sites near a treatment plant in Abruzzo (site A: hospital water; site B: domestic sewage; site C: upstream of the treatment plant; site D: activated sludge; site E: effluent). Twenty *K. pneumoniae* strains resistant to ertapenem were found at sites A, B, C, D, but not at E. These isolates were also resistant to other antibiotics: β-lactams (AMX, PIP, CTX, CAZ, CZ, cefpirome, and aztreonam, β-lactam inhibitors (AMC and piperacillin/tazobactam), fluoroquinolones (LVX and CIP), and aminoglycosides (AK, GM).

Two *K. pneumoniae* strains were found by Pellegrini et al. [43] in water samples collected from three sites (raw water-RW, water after chlorination system-CH and final effluent-FE) of WWTP located in Abruzzo. The isolates found in CH and FE were resistant to β-lactams (AMX, PIP, CF, SXT), β-lactam inhibitors (AMC), and fluoroquinolones (LVX).

### 3.2. Genotypic Evaluation

The molecular methods to characterize ARG were simple and multiplex PCR and real-time PCR.

Ten studies evaluated the genotypic profile of AMR bacteria: five studies reported only genotypic evaluation; the others reported the genotypic evaluation associated with phenotypic evaluation. Three studies [34,37,38] assessed the presence of resistance genes in the effluent wastewater of three different plants located in Piemonte (Verbania, Cannobio, and Novara). All three plants received urban wastewater, but those of Verbania and Novara also received pretreated hospital sewage.

Corno et al. [34] assessed the antibiotic resistome in the receiving water bodies (Lake Maggiore for Cannobio and Verbania, Agogna River for Novara) and in the effluent of UWWTPs. Most of the genes conferred resistance to β-lactams, including 115 different types of ARG, of which 62 (53.9%) were present in effluent wastewater. Of these, *bla*_OXA_ genes were the most abundant in effluent waters and *bla*_TEM_ genes in the receiving water bodies. However, there were also 23 ARG against TET (including 15 in receiving water bodies and effluent), 18 ARG against macrolide (including eight in receiving water bodies and effluent), and 13 ARG against aminoglycosides (including six in the effluent and two in receiving water bodies).

Di Cesare et al. [37] evaluated the abundance of the following ARG in the effluents: *tet*A (tetracyclines), *sul*II (sulfonamide), *erm*B (macrolides), *qnr*S (fluoroquinolones), and *int*1 (class I integron). The BLA resistance genes were only quantifiable in the influx of each WWTP, whereas they were positive but not quantifiable or negative in the following steps and in the effluxes. All other tested ARGs were quantifiable in all samples and all WWTPs. The genes all ranged between minimum abundances of 10^2^–10^3^ copies mL^−1^ (*sul*II: 543, *tet*A: 302, *erm*B: 189, *qnr*S: 399, *int*1: 553) up to 6 × 10^6^, 4.6 × 10^6^, for *qnr*S, *erm*B, respectively. The genes *int*1, *sul*II and *tet*A had maximum abundances of 9.8 × 10^5^, 7.1 × 10^5^, and 5.6 × 10^5^, respectively. The same authors [38] also evaluated the abundance of ARG encoding both *bla*_TEM_ (β-lactams) as well as either *tet*A, *erm*B, *qnr*S, or *int*I1 in pre-/post-disinfection effluent of each UWWTP.

Fiorentino et al. [35] assessed the impact of industrial wastewater on ARG in UWWTP of Cannobio (Piemonte). The samples were collected both under typical operating conditions with mixed urban/industrial wastewater, and with only urban wastewater. In particular, the presence of the following genes was assessed: *tet*A, *sul*II, *erm*B, *qnr*S, and *int*I1. The results showed that the origin of wastewater (industrial or urban) did not influence the resistance genes.

Subirats et al. [36] assessed the presence of ARG in the source water of an unpolluted lake (control), i.e., high quality treated wastewater intended for agricultural use (HQWR) with and without 5 μg L^−1^ CTX mixed with source water (treatment conditions). In particular, the genes *bla*_TEM_, *bla*_CTX_, *bla*_OXA_, and *bla*_KPC_ (β-lactams), and *qnr*S, *tet*A, *sul*II, *erm*B, and *int*I1, were studied. The wastewater was collected by the treatment plant that received domestic and hospital wastewater, located in Verbania. The bacterial communities exposed to HQWR showed a significantly greater abundance of *tet*A and *int*I1, while those exposed to HQWR treated with CTX did not. The genes *bla*_TEM_, *bla*_CTX-M_, *bla*_OXA_, *bla*_KPC_, *qnr*S, and *erm*B were always below the detection limit of the qPCR test, while *sul*II, *tet*A, and *int*I1 genes were found at concentrations above the detection limit.

Zanotto et al. [40] confirmed the resistance of *E. coli* to AMP and CHL through amplification of *bla*_TEM-1_ and the acetyl-transferase *cat*A1 gene.

*Int*I1 and the genes *bla*_AmpC_ and *bla*_CTX_-_M-15_ (β-lactams) were present in all isolates of *E. coli* resistant to AMP and CTX [41].

Perilli et al. [42] evaluated the presence of *bla*_KPC-3_ and *bla*_SHV-1_ (β-lactams) in all carbapenem- resistant *K. pneumoniae* strains. Only eight isolates had *bla*_TEM-1_. *Int*I1 with *aad*A2 (streptomycin) were harbored by all isolates.

Pellegrini et al. [43] showed that ten *E. coli* were positive for *bla*_AmpC_ and *bla*_TEM-1_ together, two *E. coli* for *bla*_AmpC_, and two *K. pneumoniae* were positive for *bla*_TEM-1_. *E. coli* harbored *Int*I1 with two different cassette arrays: *dfr*17-*aadA*5 (No. 2) and *aadA*10 (No. 2). The genes *dfr* and *aadA* encoded resistance to trimethoprim and streptomycin respectively and the class integron 2 with two different cassette arrays: *dfr*1-*sat*1 (No. 1) and *dfr*1-*sat*1-*aad*A1 (No. 1). The gene *sat* encodes the resistance to streptothricin.

Ferro et al. [44] evaluated the presence of three ARG, i.e., *bla*_TEM_, *qnr*S, and *tet*W (tetracyclines), isolated by intracellular (resistant *E. coli*) and total DNA (whole water suspension). The *bla*_TEM_ *qnr*S, and *tet*W genes were detected in intracellular DNA, but were absent or present at undetectable concentrations in total DNA.

### 3.3. Wastewater Treatment Processes

The selected scientific studies showed that the main disinfection systems involve the use of peracetic acid (PAA), chlorine, and ultraviolet (UV) radiation.

Two articles [34,38] compared resistance genes from disinfection systems in three different UWWTPs (Verbania, Novara, and Cannobio) located in Piemonte. In particular, final disinfection was performed with sodium hypochlorite (0.05 mg L^−1^ for 45 min) in Verbania, PAA (0.010 mg L^−1^ for 55 min) in Cannobio, and UV-C lamps (25.8 mJ cm^−2^ for 2 s) in Novara. The results of Corno et al. [34] suggested that controls were needed on effluents to limit their spillage into surface waters and, consequently, reduce the concentration of ARG in the environment, at least until more effective treatments were available. Di Cesare et al. [38] showed a significant reduction of ARG (*tet*A, *bla*_TEM_, *qnr*S, *erm*B, and *int*I1) from influent to effluent waters (post-disinfection) of 97%, 85%, and 84% by disinfection with sodium hypochlorite in Verbania, UV-C lamps in Novara, and PAA in Cannobio, respectively.

Another study [39] evaluated the efficacy of three different disinfection systems in three UWWTPs located in Milan on *E. coli* strains resistant to AMP, CHL, and TET. The authors showed a high sensitivity of *E. coli* to PAA (2 mg L^−1^ for 45 min) for WWTP1 and depending on the UV dose in WWTP2. In particular, for UV doses of 50–80 mJ cm^−2^ (e.g., for the effluent discharged into surface water), the inactivation is about one order of magnitude, and for UV doses of 150–300 mJ cm^−2^ (e.g., for agricultural reuse), it is three orders of magnitude. In WWTP3, sodium hypochlorite (1.8 mg L^−1^ for 35 min) showed strong efficacy against *E. coli*, with a reduction of three orders of magnitude.

In vitro experimental studies conducted at different concentrations of AMP [40] showed that PAA treatment is able to reduce the amount of AMP-resistant *E. coli* to within the limits of *E.coli* for irrigation use (≤10 CFU/100 mL) [50]. In particular, at the lowest in vitro concentration of AMP (8 μg ml^−1^), the percentage of resistant *E. coli* was significantly reduced (37.2% pre- vs. 3% post-disinfection). In contrast, Fiorentino et al. [35] stated that PAA did not influence the removal of ARG, and was ineffective in controlling the environmental spread of resistance genes.

Rizzo et al. [24] evaluated the effects of UV radiation treatment vs. chlorination on two resistant *E. coli* strains. The UV disinfection process resulted in total inactivation of resistant *E. coli* after 60 min of irradiation (1.25 × 10^4^ μW s cm^−2^), while chlorine disinfection (2 mg L^−1^ of active chlorine) was equally effective after 120 min. Chlorination had no effect on the change in resistance for AMX, CIP, and SMZ, while UV treatment only resulted in a decrease in CIP resistance after 60 min of treatment.

Ferro et al. [44] showed the effect of UV radiation combined with H_2_O_2_ disinfection at different treatment times (T0, T30, T120, and T240 min) on the inactivation of *E. coli* resistant to AMP, CIP, TET, and ARG (*bla*_TEM_, *qnr*S, and *tet*W). Antibiotic-resistant *E. coli* survived after 60 min of disinfection treatment. After this treatment, a decrease of *bla*_TEM_ and *qnr*S genes was observed in intracellular DNA, while *tet*W was still present after 90 min of treatment. On total DNA, *bla*_TEM_ and *tet*W genes were below the detection limit, and the *qnr*S gene was present at all four treatment times.

In the last decade, the effectiveness of solar radiation has also been considered. Rizzo et al. studied the effect of chlorine treatment compared with the effects of solar radiation [46] and UV radiation [24] on two strains of *E. coli* resistant to AMX, CIP, and SMZ. Solar radiation resulted in poor inactivation of resistant *E. coli* strains after 180 min, as did a chlorination process (2 mg L^−1^ of active chlorine) after 60 min. Chlorination had no effect on the change in resistance for all three antibiotics, while treatment with solar radiation resulted in a decrease in CIP resistance after 180 min of treatment. The UV irradiation (1.25 × 10^4^ μW s cm^−2^) after 60 min resulted in the total inactivation of resistant *E. coli*, while disinfection with chlorine (2 mg L^−1^ of active chlorine) achieved the same result after 120 min. The same authors [47] also assessed the efficacy of new treatments such as the TiO_2_-simulated photocatalysis solar process and the natural solar photocatalytic process on *E. coli* resistant to CIP, CEF, TET, and VAN selected by the effluent (activated sludge). Only the simulated photocatalysis solar process (0.05 g TiO_2_ L^−1^ in suspension) inactivated the resistant *E. coli* after 60 min of exposure.

Recently, Fiorentino et al. [47] studied new treatments including solar disinfection and solar-driven advanced oxidation processes (H_2_O_2_/sunlight, TiO_2_/sunlight, TiO_2_/H_2_O_2_/sunlight, photo-solar Fenton) on *E. coli* resistant to AMP, CIP, and TET. The main processes that led to high inactivation rates of *E. coli* were H_2_O_2_/TiO_2_/sunlight and H_2_O_2_/sunlight. None of the processes, however, influenced the antibiotic resistance of the surviving colonies. To evaluate the bacterial regrowth potential of *E. coli*, the treatment process with H_2_O_2_/sunlight (50 mg L^−1^) was compared with classic disinfection with active chlorine (1 mg L^−1^ of active chlorine). Chlorine disinfection was more effective than H_2_O_2_/sunlight in the total inactivation of MDR *E. coli* (15 min vs. 90 min) but less effective in controlling their regrowth (24 h *vs.* 48 h). Subsequently, Fiorentino et al. [48] evaluated the effects of sunlight on the inactivation of resistant *E. coli* and Enterococci, showing a decrease in the percentage of inactivation in *E. coli* compared with Enterococci (25.2% vs. 12.6% at 240 min of exposure).

## 4. Summary, Knowledge Gaps, and Conclusions

This review reports some important contributions made in the last decade on AMR in the aquatic environment, to evaluate both the spread of ARB and ARG in UWWTPs and the effectiveness of some treatment techniques.

Antibiotics are the most successful drugs used for human and veterinary therapy. However, large quantities of antibiotics are released into urban wastewater because of incomplete metabolism in humans or the disposal of unused antibiotics, which find their way into various environmental settings. The emergence and rapid spread of ARB has led to growing concern for public health. ARB and ARG have been widely detected in wastewater samples [51].

To reduce the frequency of infections by AMR microorganisms associated with hospital and community health care, Italy has proposed the first National Plan against AMR (PNCAR 2017–2020) with an agreement between the Government, the Regions, and the Autonomous Provinces of Trento and Bolzano. The objective of this PNCAR is to monitor the antibiotic resistance of a selected group of bacterial species isolated from infections of clinical relevance.

This literature review revealed a large number of studies carried out in five regions in Italy: Campania (No. 6), Piemonte (No. 5), Abruzzo (No. 3), Lombardia (No. 2), and Sicilia (No. 1). It is concluded that national scientific commitment is necessary for the evaluation of the AMR from wastewater. One aspect to highlight is that many researchers do not clearly indicate the origin of the wastewater studied (influent or effluent). These data are very important to determine the quality of the effluents that spill into surface waters and the effects of their reuse for irrigation purposes.

Although a few studies have assessed the presence of *K. pneumoniae* and Enterococci in wastewater, resistance against certain classes of antibiotics is more prevalent. In particular, *K. pneumoniae* is mainly resistant to AMX, PIP, ETP, AMC, and LVX; *E. coli* to AMP, TET, and CIP; and Enterococci to AMP and TET.

Most of the examined articles were focused on *E. coli*, because it is considered both an indicator of fecal contamination and a possible indicator to evaluate the spread of AMR in the aquatic environment [52].

The literature provides little information on antibiotic-resistant heterotrophic and non-coliform bacteria [53]. These microorganisms are present in activated or waste sludge, and could play an important role in the development and transmission of AMR. Finally, some studies highlight the spread of resistant Enterococci in non-hospital environments that are not completely eliminated by most conventional wastewater treatment processes, and neither Italian nor European legislation provides for limits for their concentration [49].

Resistance to β-lactams can be explained by their widespread use in the treatment of human or animal bacterial infections [40].

Among the ARG, the most common are those that code for resistance to β-lactams (*bla*), for TET (*tet*), erythromycin (*erm*B), quinolones (*qnr*S), and sulfonamides (*sul*). Integron class 1 is the most common in wastewater. A genotypic characterization confirmed the results obtained from the phenotypic evaluation.

Among known wastewater treatment systems, sodium hypochlorite, PAA, and UV radiation are the main control systems for ARB and ARG. The effects of different disinfection processes depend on the disinfectant and its applied dose.

Although numerous studies have been published on this topic, no conclusions can be drawn on the role of UWWTPs in the spread of AMR [38,40]. To date, it is not possible to indicate the role of wastewater treatment plants in the spread of AMR, nor to understand the effectiveness of common strategies to limit AMR, since water quality, local regulations and restrictions, process costs, type of crop, irrigation method, soil type, and environmental footprint all affect the efficacy of wastewater treatment [54]. At present, there is no single optimal advanced treatment process; therefore, some researchers are evaluating various combinations of unit treatment processes [55]. However, some alternatives for efficient ARG removal from effluent of wastewater treatment plants have been recently proposed, including the use of synthesized hydrated manganese oxide coupled with permanganate, the metal-free photocatalyst graphitic carbon nitride (g-C_3_N_4_), and the natural zeolite clinoptilolite enriched with silver (AgNZ) [56,57,58]. In addition, bacterial resistance to commonly used disinfectants is often overlooked [59].

In the future, researchers dealing with wastewater should investigate the effectiveness of disinfection methods to produce effluents with a limited presence of ARB and ARG because these resistance forms could move from the environment to humans, causing risks to human health.

## Figures and Tables

**Table 1:** Overview of antimicrobial resistance in wastewater in Italy, divided by geographical area.

**References**	**Wastewater Location**	**Disinfection Treatment**	**Studied** **Bacteria**	**Resistance** **Genes**	**Main** **Outcomes**
Corno G. et al., 2019 [34]	Verbania, Cannobio, Novara (Piemonte)	chlorine in Verbania, peracetic acid in Cannobio, UV radiation in Novara		Resistome of the bacterial communities	The release of uncontrolled dilutions of effluents into surface water represents a potential threat to the environment.
Fiorentino A. et al., 2019 [35]	Cannobio (Piemonte)	peracetic acid		*tet*A, *sul*2, ermB, qnrS and class 1 integron (*int*I1)	The overall removal efficiencies in terms of absolute gene concentrations were 9% (*int*I1), 10% (*tet*A), 14% (*sul*2), 8% (*qnr*S), and 8% (*erm*B).
Subirats J. et al. 2019 [36]	Verbania (Piemonte)	chlorine		*bla*_TEM_, *bla*_CTX-M_, *bla*_OXA_ and *bla*_KPC_, *qnr*S, *tet*A, *sul*II, *erm*B, *ars*B and *czc*A, and class 1 integron (*int*I1)	The use of high-quality treated wastewater for agricultural purposes can cause changes in natural microbial communities and resistance genes.
Di Cesare A. et al., 2016 [37]	Verbania, Cannobio, Novara (Piemonte)	chlorine in Verbania, peracetic acid in Cannobio, UV radiation in Novara		*tet*A, *sul*II, *bla*_TEM_, *bla*_CTXM_, *erm*B, *qnr*S and class 1 integron (*int*I1)	Removal efficiencies in terms of absolute gene concentrations were 97% (chlorine treatment), 85% (UV radiation) and 84% (peracetic acid treatment).
Di Cesare A., et al. 2016 [38]	Verbania, Cannobio, Novara (Piemonte)	chlorine in Verbania, peracetic acid in Cannobio, UV radiation in Novara		*tet*A, *erm*B, *bla*_TEM_, *qnr*S, and class 1 integron (*int*I1)	Peracetic acid and chlorine disinfection promote the selection of ARGs and horizontal gene transfer.
Turolla A. et al., 2018 [39]	Milano (Lombardia)	chlorine, UV radiation, peracetic acid	*E. coli*		Different influence of various disinfectants on resistant *E.coli*
Zanotto C. et al., 2016 [40]	Milano (Lombardia)	peracetic acid	*E. coli*	*bla* and *cat*	Peracetic acid was very effective in reducing *E.coli.*
Piccirilli A. et al., 2019 [41]	L’Aquila (Abruzzo)		*E. coli*	*bla*_KPC_, *bla*_TEM_, *bla*_SHV_, *bla*_CTX-M_, *bla*_OXA-48_, *bla*_OXA-23_, *bla*_AmpC_, *bla*_VIM_, *bla*_IMP_, *bla*_NDM_, *bla*_CphA_	The presence of AMR bacteria and resistance genes highlight the poor efficacy of biological and chemical-physical processes in treatment plants.
Perilli M. et al. 2013 [42]	L’Aquila (Abruzzo)		*K. pneumoniae*	*bla*_KPC_, *bla*_OXA-48_, *bla*_NDM_, *bla*_VIM_, *bla*_IMP_, *bla*_TEM_, *bla*_SHV_, *bla*_CTX-M_ and class 1 integron (*int*I1)	Both environmental and clinical *K. pneumoniae* showed a multidrug resistant phenotype and the presence of some mobile genetic elements.
**References**	**Wastewater Location**	**Disinfection Treatment**	**Studied** **Bacteria**	**Resistance** **Genes**	**Main** **Outcomes**
Pellegrini C. et al., 2011 [43]	L’Aquila (Abruzzo)	chlorine	*E. coli* *K. pneumoniae*	Class 1 and 2 integron	Presence of *Enterobacteriaceae* carrying class 1 and 2 integrons.
Ferro G. et al., 2016 [44]	Salerno (Campania)	UV/H_2_O_2_ process	*E. coli*	*bla*_TEM_, *qnr*S, *tet*W	The UV/H_2_O_2_ process may not be an effective disinfection process to limit the spread of ARGs.
Rizzo L. et al., 2014 [45]	Salerno (Campania)	Comparison among simulated TiO_2_ solar photocatalysis (SSP) and the natural solar photocatalytic process (SP)	*E. coli*		Total inactivation of *E. coli* after 60 min total irradiaton time was observed for SSP, while SP process did not result in total bacteria inactivation.
Rizzo L. et al., 2013 [24]	Salerno (Campania)	Comparison among chlorine and UV radiation	*E. coli*		UV radiation treatment has an effect on antibiotics and *E. coli* strains after 60 min of irradiation compared to 120 min chlorine contact.
Rizzo L. et al., 2012 [46]	Salerno (Campania)	Comparison among chlorine and solar radiation	*E. coli*		Solar radiation resulted in poor inactivation compared to the chlorination process.
Fiorentino A. et al., 2015 [47]	Salerno (Campania)	Comparison among H_2_O_2_/sunlight, TiO_2_/sunlight, H_2_O_2_/TiO_2_/sunlight, solar photo-Fenton and chlorine	*E. coli*		Among the innovative processes, treatments with H_2_O_2_/TiO_2_/sunlight and H_2_O_2_/sunlight have shown good efficacy.
Fiorentino A. et al., 2017 [48]	Salerno (Campania)	Solar radiation	*E. coli* Enterococci		Approximately 1 log inactivation of resistant *E. coli* and Enterococci after 4 h solar radiation.
Russo N. et al., 2019 [49]	San Michele di Ganzaria (Sicilia)		Enterococci		WWTP do not completely eliminate resistant Enterococci.
